# MBFFNet: Multi-Branch Feature Fusion Network for Colonoscopy

**DOI:** 10.3389/fbioe.2021.696251

**Published:** 2021-07-14

**Authors:** Houcheng Su, Bin Lin, Xiaoshuang Huang, Jiao Li, Kailin Jiang, Xuliang Duan

**Affiliations:** ^1^College of Information Engineering, Sichuan Agricultural University, Ya’an, China; ^2^College of Science, Sichuan Agricultural University, Ya’an, China

**Keywords:** multi-branch feature, fusion network, colonoscopy, medical image segmentation, MBFFNet

## Abstract

Colonoscopy is currently one of the main methods for the detection of rectal polyps, rectal cancer, and other diseases. With the rapid development of computer vision, deep learning–based semantic segmentation methods can be applied to the detection of medical lesions. However, it is challenging for current methods to detect polyps with high accuracy and real-time performance. To solve this problem, we propose a multi-branch feature fusion network (MBFFNet), which is an accurate real-time segmentation method for detecting colonoscopy. First, we use UNet as the basis of our model architecture and adopt stepwise sampling with channel multiplication to integrate features, which decreases the number of flops caused by stacking channels in UNet. Second, to improve model accuracy, we extract features from multiple layers and resize feature maps to the same size in different ways, such as up-sampling and pooling, to supplement information lost in multiplication-based up-sampling. Based on mIOU and Dice loss with cross entropy (CE), we conduct experiments in both CPU and GPU environments to verify the effectiveness of our model. The experimental results show that our proposed MBFFNet is superior to the selected baselines in terms of accuracy, model size, and flops. mIOU, *F* score, and Dice loss with CE reached 0.8952, 0.9450, and 0.1602, respectively, which were better than those of UNet, UNet++, and other networks. Compared with UNet, the flop count decreased by 73.2%, and the number of participants also decreased. The actual segmentation effect of MBFFNet is only lower than that of PraNet, the number of parameters is 78.27% of that of PraNet, and the flop count is 0.23% that of PraNet. In addition, experiments on other types of medical tasks show that MBFFNet has good potential for general application in medical image segmentation.

## Introduction

Medical image processing is an important part of medical processes. At present, the main research directions in medical image processing include image segmentation, structure analysis, and image recognition. Among these, image segmentation is very important for the detection of lesions and organs, which significantly aids the development of medical automation, reduces the burden on medical workers, and reduces the incidence of medical accidents caused by human error (Litjens et al., 2017). In 2018, there were an estimated 4.8 million new cases of gastrointestinal (GI) cancers and 3.4 million related deaths worldwide. GI cancers account for 26% of the global cancer incidence and 35% of all cancer-related deaths (Arnold et al., 2020). Endoscopy is the gold standard for GI examinations (Deeba et al., 2019; Li et al., 2021). Gastroscopy is an examination of the upper digestive tract, which includes the esophagus, stomach, and the first part of the small intestine, whereas colonoscopy covers the large intestine (colon) and rectum. Both tests involve the real-time viewing of the GI tract using a digital high-definition endoscope. Endoscopy is resource-intensive and requires expensive technical equipment and trained personnel (Pogorelov et al., 2017). Both endoscopy and the removal of potentially pre-cancerous lesions are essential for the prevention of colorectal cancer. The semantic segmentation method of artificial intelligence can be used to assist colonoscopy detection, which can significantly reduce the risk of misjudgment and the omission of medical workers for various reasons, resulting in polyp canceration, colorectal tumor lesions, and colorectal cancer from early to late stages, as well as delayed treatment (Akbari et al., 2018). It is thus important to achieve early prevention, early detection, and early treatment. A large number of experimental studies have shown that early colonoscopy can reduce the incidence of colorectal cancer by 30% (Haggar and Boushey, 2009). In clinical medical treatment, the accurate real-time segmentation of polyps is a challenging task. First, the same type of polyp may be due to different stages of colorectal cancer and may have a different constitution. In addition, there may be different sizes, shapes, and colors, which affects the actual segmentation result (Nguyen et al., 2020). Second, because polyps and surrounding mucosa possess similar characteristics, it is difficult to segment the boundary clearly, and commonly employed segmentation method cannot obtain ideal segmentation results (Ganz et al., 2012; Bernal et al., 2014). Third, owing to the specific nature of medical images, it is often difficult to achieve high accuracy and fast speed simultaneously. Therefore, commonly used medical image segmentation model often ignores the size of the model while ensuring accuracy, resulting in an oversized model and slow segmentation speed; it is thus unable to provide real-time segmentation for colonoscopy (Bernal et al., 2012, 2015). Therefore, in medical automation and to achieve the early prevention of colorectal cancer, it is important to propose a method that segments polyps with sufficient accuracy to prevent the missed detection of polyps and to ensure that the model will not be too bloated, leading to slow speed.

Based on the machine algorithm of manually extracted features, features such as color, shape, and appearance have been applied to the classifier to detect polyps (Armato et al., 2017). Because of the limitation of the expression ability of manually extracted features, sufficient features cannot be effectively obtained for classifier classification (Breier et al., 2011), and there is a high rate of missed detection, which cannot be effectively applied to accurately segment polyps. However, based on the depth study of the semantic segmentation method of the polyp segmentation method, there has been good progress so far. Armato et al. (2017) used the FCN8 (Long et al., 2015) semantic segmentation model to split polyps, but because FCN8 cannot effectively retain low-dimension detail characteristics, it cannot effectively segment polyps and membranes around the border, so the use of FCN8 polyp segmentation is mistakenly identified and residual (Xia, 2020). Other semantic segmentation models are applied to life scenarios, such as PSPNET (Zhao et al., 2017), and although they use a feature pyramid, retain as many low-dimensional features as possible, and improve the boundary extraction effect of FCN8, they still fail to meet the requirements of precision medicine. Meanwhile, other models, such as Deeplabv3 (Chen et al., 2017), Deeplabv3+ (Chen et al., 2018), LinkNet (Chaurasia and Culurciello, 2018), and FPN (Lin et al., 2017), all have similar problems. In UNet (Ronneberger et al., 2015), UNet++ (Zhou et al., 2018, 2020), ResUNet++ (Jha et al., 2019), and U^2^Net (Qin et al., 2020), which are medical image segmentation models, the adoption of more detailed features has a good effect on the polyp boundary segmentation, but these methods with the characteristics of the UNet (Ronneberger et al., 2015) method to keep figure overlay information, model and quantity, and flop count are inevitable. In real-time polyp segmentation, there is still a disadvantage in that it is unable to meet real-time requirements. Fang et al. (2019) proposed a three-step selective feature aggregation network with area and boundary constraints, which was applied to the precise segmentation of polyps. Because the relationship between the area and boundary was considered in this network, excellent segmentation results were obtained. However, the PraNet (Fan et al., 2020) model proposed by Fan et al. (2020) adopted the reverse attention method and achieved excellent results in polyp segmentation. However, it aimed to achieve segmentation that was too precise, resulting in a large flow count, which could not be applied to general computer applications and could not be popularized on a large scale.

In this study, to better achieve the precise real-time segmentation task of polyps and considering these problems, we developed the following strategies:

(1) Avoid the loss of local low-dimensional features by large up-sampling directly, which leads to the loss of too many features on the segmenting boundary and the inability to restore complete edge information.
(2) Avoid superimposing feature information on channel dimensions only through feature maps to retain feature information, which will lead to an overbloated feature map in the last few layers of the feature map, resulting in the model requiring a large number of calculations.

Based on these strategies, we propose a multi-branching feature fusion network for polyp segmentation. We first propagated the context information to the higher-resolution layer through progressive up-sampling to obtain the preliminary polyp features. This method followed strategy 1, and we avoided the channel dimension superposition feature information of the UNet (Ronneberger et al., 2015) series-related models, and selected the method of feature graph multiplication to fuse features, which followed strategy 2. Thus, most of the feature information was well retained, and the boundary information could be obtained effectively. The accuracy is equal to that of UNet (Ronneberger et al., 2015), and the flop count was effectively reduced. Then, through the feature information of another branch, the concat method was adopted to provide more detailed low-dimensional feature information as a complement for feature fusion in order to ensure that the accuracy is slightly better than that of UNet++ (Zhou et al., 2018, 2020), ResUNet++ (Jha et al., 2019), and other networks, whereas the actual running speed is much better than other models; in addition, it has the advantages of high training efficiency and strong generalization ability. This study makes the following contributions:

(1) We propose a model improvement approach that provides effective support for the efficient application of deep learning models in large-scale medical environments.
(2) An efficient polyp segmentation network is proposed that can accurately and effectively segment polyp images without the need for costly computer resources. Real-time colonoscopy detection can be guaranteed using existing computer resources.

Our proposed model shows good performance and generalization ability in a variety of different medical image datasets and can be extended to the detection of other medical issues.

In this article, the detailed model structure and parameter number verification are described in section “Materials and Methods,” the experimental part of the model is discussed in section “Experiments,” and a summary of the model is presented in section “Conclusion.”

## Materials and Methods

In this section, we first introduce and analyze the advantages and disadvantages of PspNet (Zhao et al., 2017) and UNet (Ronneberger et al., 2015) models, and we make a detailed comparison with this model to provide a better understanding of our multi-branch feature fusion network (MBFFNet).

### Baseline

**FIGURE 1:**
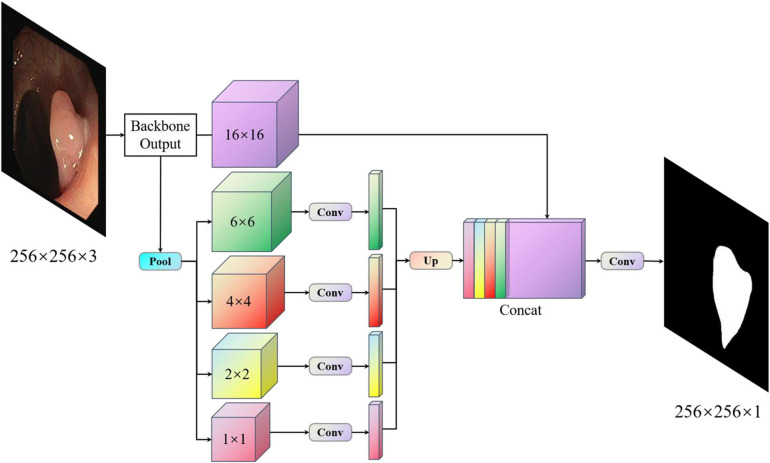
Pspnet structure.

With PspNet (Zhao et al., 2017), researchers believe that the existing models have segmentation errors owing to insufficient context information and global information under different receptive fields. PspNet model structure diagram as shown in Figure 1. Therefore, a hierarchical global priority containing information of different scales between different subareas is proposed, which is called the pyramid pooling module (Zhao et al., 2017). Four features of different pyramid scales are integrated, from the roughest feature in the first-row global pooling to a single output, and the next three are pooling features of different scales. After each level, a 1 × 1 convolution is used to reduce the level channel to the original 1/N. Then, it is converted to the pre-pooled size through bilinear interpolation, and finally, concatenation is carried out. In this way, the global features are obtained, the global information of different receptive fields is obtained, and good semantic segmentation results are obtained. However, as the pooled information of different scales is directly converted to the dimensions before pooling by an up-sampling method, the feature loss of the model is relatively large in the low-dimensional features. For medical image segmentation requiring accurate boundary results, although PspNet (Zhao et al., 2017) has a good overall effect, it is not suitable for application in medical image segmentation because of its incomplete retention of fine edge features and the inability to obtain complete boundary results.

**FIGURE 2:**
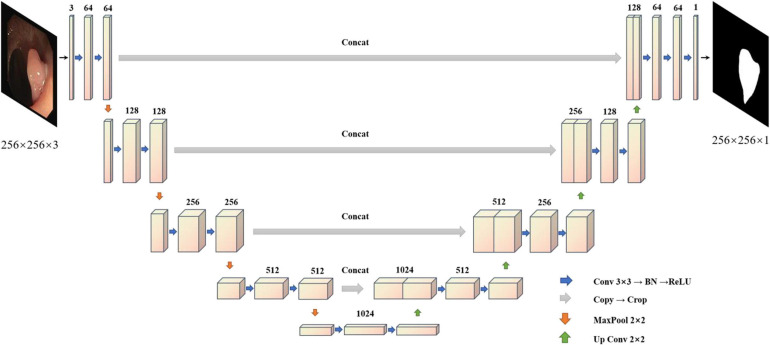
Unet structure.

To solve the problem of medical image segmentation and accurate boundary segmentation, UNet (Ronneberger et al., 2015) employs a completely different method of feature fusion. UNet uses VGG16 (Simonyan and Zisserman, 2014) as the backbone network backbone, and through the different location of the backbone for the characteristics of the different size chart, on the four double sampling, and after each sampling on a layer to obtain the characteristics of the figure for Mosaic, UNet (Ronneberger et al., 2015), researchers in order to retain more features, will feature in the channel dimension stitching together, forming thicker characteristics (Simonyan and Zisserman, 2014; Ronneberger et al., 2015). It is used in the same phase in the jumping connections, rather than to directly supervise and experiences loss with respect to high-level semantic features. These characteristics of a graph are a combination of more low-level image edge features and features with different scales, so the multi-scale prediction can be performed, making the model on the edge of the segmentation image restoration have more detailed information. However, because UNet (Ronneberger et al., 2015) employs the channel dimension splicing characteristic figure, combining to form the characteristics of the figure will result in many similar repeated characteristics, and characteristics of the severe figure redundancy phenomenon are costly in later calculations, requiring a large number of calculations and a high flop count, which affects the speed of the model. The model diagram of UNet (Ronneberger et al., 2015) is shown in Figure 2.

### MBFFNet

**FIGURE 3:**
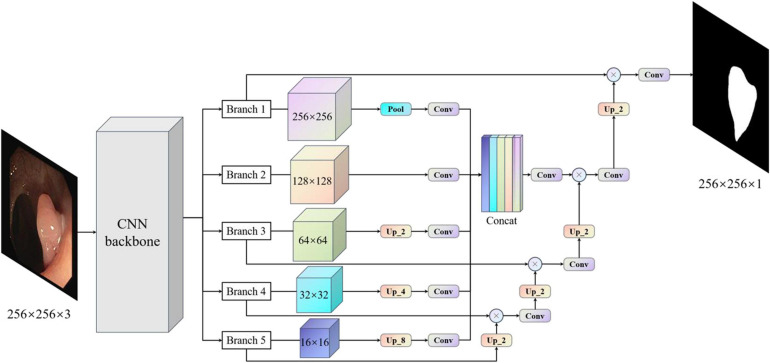
Multi-branch feature fusion network. The backbone initially extracts the features of images, and images with different subsampling multiples are superimposed in an hourglass image pyramid (subsample images have a size larger than 128, and up-sample images have a size smaller than 128, which is equal to 1 × 1 standard convolution for images with size larger than 128). To maximize the use of cross-channel and cross-resolution image branches, each branch is up-sampled and multiplied by the previous layer. Finally, the predicted image of the original image size is obtained.

Considering the above problems and the advantages and disadvantages of different models, we proposed the MBFFNet, which has a better lightweight network structure, and can simultaneously consider model accuracy and rapid deployment. Compared with UNet (Ronneberger et al., 2015) and its derivative versions, MBFFNet has better accuracy and requires fewer computations. In order to better validate the model of the network segmentation effect, we adopted the same approach as UNet (Ronneberger et al., 2015), with the VGG16 (Simonyan and Zisserman, 2014) network as the backbone, and multiple branch feature fusion network using the U-shaped structure of the UNet (Ronneberger et al., 2015) framework. We selected the Relu activation function to ensure that the model can reduce the flop count, and we abandoned the UNet (Ronneberger et al., 2015) channel dimension of the connection method. MBFFNet did not choose the method of FCN8 (Long et al., 2015) feature combination and fusion, but chose the method of feature multiplication for feature fusion. Therefore, there are two important advantages: (1) it avoids the burden of excessive computation owing to the excessive feature channels caused by the direct Mosaic of feature graphs; and (2) as the number of network layers increases, overfitting is easily caused, but considering that the use of feature information between the upper and lower layers can solve this problem well. We weighted the normalized weight to the features of each pixel of the next layer through a dot product operation. This is no longer an attention mechanism based on channels, but an attention mechanism based on the pixel level (Jie et al., 2018). However, it is inevitable that low-dimensional feature information will be lost to a certain extent. Although the loss of such low-dimensional feature information is not serious after our experiment, the loss of some low-dimensional feature information may prevent the segmentation of a complete and detailed boundary image during the precise boundary division of polyps. Therefore, after using the original U-shaped structure, our model maximizes the characteristics of the five branches in the figure. Through pooling, without processing, the bilinear interpolation method is used for samples of the same size two/four/eight times. This will be an hourglass-like combination that will sample the functional layers at different times and then add low-dimensional edge feature information through convolution after the channel dimension concat has passed, adding second information to integrate features with other maps, a complete multi-branch feature fusion model network structure diagram, as shown in Figure 3. In this way, we can ensure that the information of low-dimensional features is preserved as much as possible and avoid the loss of low-dimensional features caused by the direct use of single up-sampling. The continuous pixel-based attention mechanism makes the model more precise in the segmentation of image edges and other information. At the same time, it also avoids the excessive pursuit of keeping feature information of different scales as far as possible in UNet (Ronneberger et al., 2015), which adopts feature graphs to add channel dimensions, resulting in too many channels, the need for too many calculations, and increased computer burden.

## Experiments

### Dataset

The polyp images used in this section were derived from the following datasets: ETIS, CVC-ClinicDB, CVC-ColonDB, Endoscene, and Kvasir. Kvasir is the largest and most extensive dataset released in 2017, and we selected polyp images from a subcategory of the Kvasir dataset (polyps). CVC-ClinicDB, which is also known as CVC-612, consists of 612 open-access images from obtained 31 colonoscopy clips. The CVC-ColonDB is a small database containing 380 images from 15 short colonoscopy sequences. ETIS is an established dataset containing 196 images of polyps for the early diagnosis of colorectal cancer. Endoscene is a combination of CVC-612 and CVC300. We integrated these data and eliminated the fuzzy images and finally obtained 1450 polyp images as the experimental data in this section.

To prove that the proposed model has better generalization ability, we collected a variety of medical image segmentation datasets for verification of our model. Common medical images share certain similarities. Therefore, we selected a larger number of medical image datasets to verify the robustness of our model.

In addition, our datasets are obtained from publicly available competitive medical datasets online, follow standard biosecurity and institutional safety procedures, and can be downloaded online. The raw data are available in articles, supplements, or repositories.

#### Corneal Nerve Dataset

This dataset consists of 30 images from the subbasal corneal nerve plexus obtained in normal and pathological subjects. Thirty images were obtained from 30 different normal or pathological subjects (diabetes mellitus, pseudoextirpation syndrome, and keratoconus). The instrument used to acquire these data was a Heidelberg Retina Tomograph II with a Rostock Corneal Module (HRTII32-RCM) confocal laser microscope.

#### Liver Dataset

This dataset was provided by the MICCAI 2018 LITS Challenge and consisted of 400 CT scans. Two distinct labels were provided for ground truth segmentation: liver and lesion. In our experiment, we treated only the liver as positive and the other parts as negative.

#### Lung Dataset

This dataset was provided by the Lung Image Database Consortium Image Collection (LIDCIDRI) and was collected by seven academic centers and eight medical imaging companies. To simplify the processing, only the lungs were segmented, and the remaining non-lung organs were treated as the background.

#### Electron Microscopy (EM) Dataset

This dataset was provided by the electron microscopy (EM) Segmentation Challenge as part of ISBI 2012. The dataset consisted of 30 (512 × 512 pixels) continuous slice transmission electron microscope images of the ventral nerve cord of the first instar larvae of *Drosophila melanogaster*. Referring to the example in Figure 3, each image has a corresponding fully annotated base-instance split map of the cell (white) and cell membrane (black).

#### Neums Dataset

The dataset was provided by the HE Data Science Bowl 2018 Segmentation Challenge and consisted of 670 segmenting nuclear images from different patterns (bright and fluorescent). This is the only dataset in this work that uses instance-level annotation, where each kernel is colored differently.

#### Ocular Vascular Dataset

This task is based on the DRIVE dataset, which uses photographs from the diabetic retinopathy screening program in Netherlands. The aim was to isolate the blood vessels in the fundus image.

#### Dataset of Esophageal Cancer

This dataset was provided by the First Affiliated Hospital of Sun Yat-sen University and comprised a total of 13,240 CT images (80 × 80) labeled by professional doctors. The goal of this dataset was to segment the esophageal cancer region in the CT image, with the non-esophageal cancer region as the background.

### Experimental Setting

#### Environment

For the polyp segmentation experiment in this section, the framework used for the training model was TensorFlow (Abadi et al., 2016). Using the ADAM optimizer, the initial learning rate was set to 0.001. The experiment was carried out on a platform with an Intel (R) Xeon (R) Silver 4208 CPU at 2.10 GHz, 2.10 GHz (two processors), 64.0 GB RAM, Windows 64-bit operating system, NVidia Titan V graphics card, and 12 GB video memory capacity. In actual production, we can choose a better lightweight backbone, such as GhostNet (Han et al., 2020) and MobileNetv3 (Howard et al., 2017, 2020; Sandler et al., 2018).

#### Data Enhancement

Considering the polyps, liver, bowel, and medical images compared to natural images, medical imaging has the following characteristics. First, compared to a variety of modes, different imaging mechanisms of different modal medical images also have different characteristics, such as format, size, and quality, and it is necessary to better design the network to extract features of different modes. Second, the shape, size, and position of different tissues and organs vary greatly. Third, the texture feature is weak and requires a higher feature extraction module. Fourth, the boundary is fuzzy, which is not conducive to accurate segmentation.

To train our model effectively, we divided the dataset into an 8:2 ratio. Eighty percent of the datasets were used for model training and 20% for model testing.

**FIGURE 4:**
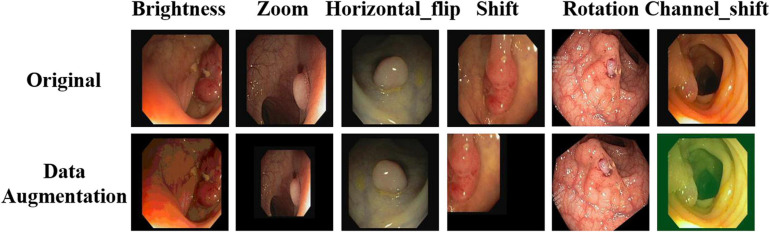
Image enhancement renderings.

To improve the robustness of the model, appropriate image enhancement is required for the training image. In this study, brightness enhancement, scaling, horizontal flip, shift, rotation, and channel transformation were performed on the training image. Owing to the limited number of medical images, we could not use the limitation of commonly used image tasks, so we chose the most commonly used data enhancement parameters of existing medical images. The specific proportions and effects are listed in Table 1 and Figure 4, respectively.

**TABLE 1:** Image enhancement setting parameters.

**Operation**	**Proportion**
Brightness	−0.2 to 0.2
Zoom	−0.75 to 2
Horizontal flip	0.5
Shift	0.5
Rotation	−0.5 to 0.5
Channel transformation	10

#### Accuracy Evaluation Index

To fully verify the accuracy of the proposed model, we chose three evaluation indicators to evaluate the model as a whole in order to more fully and intuitively prove the effect of our model. Three metrics are as follows.

##### mIOU

This calculates the ratio of the intersection and union of two sets of true and predicted values. This ratio is the sum of true positive (TP) divided by TP, false positive (FP), and false negative (FN). FN indicates that the prediction was negative, but the label result was positive; an FP is actually a negative case, and for a TP, the prediction is positive. In fact, it is also a positive example, indicating that the prediction result is correct, where *p*_*ij*_ represents the number of real values and is predicted to be *j*, and *k*+1 is the number of classes (including the background). *P*_*ii*_ is the number of values predicted correctly, and *p*_*ij*_, and *p*_*ji*_ represent FP and FN, respectively (Kingma and Ba, 2015). The formula for calculating mIOU is as follows:

(1) $$\text{mIOU}=\frac{1}{k+1}\,\sum_{i=0}^{k}\frac{p_{i\,i}}{\sum_{j=0}^{k}p_{i\,j}+\sum_{j=0}^{k}p_{j\,i}-p_{i\,i}}$$

##### F score

In an ideal situation, it would be best if both evaluation indexes were high. However, a high precision generally means low recall, and high recall means low precision. Therefore, in practice, it is often necessary to make a trade-off according to specific circumstances, such as the general search situation. To ensure the recall rate, the precision rate should be improved as much as possible. For example, for cancer detection, seismic detection, financial fraud, and so on, the recall rate should be increased as much as possible to ensure accuracy. A new index, the *F* score, is derived, which comprehensively considers the harmonic value of precision and recall (Flach and Kull, 2015). The calculation formula is as follows:

(2) $$P\,r\,e\,c\,i\,s\,i\,o\,n=\frac{T\,P}{T\,P+F\,P}+\text{FP}$$

(3) $$R\,e\,c\,a\,l\,l=\frac{T\,P}{T\,P+F\,N}$$

(4) $$F-\mathrm{score}=\left(1+\beta^{2}\right)\cdot \frac{P\,r\,e\,c\,i\,s\,i\,o\,n\cdot R\,e\,c\,a\,l\,l}{\beta^{2}\cdot P\,r\,e\,c\,i\,s\,i\,o\,n\,+\,R\,e\,c\,a\,l\,l}$$

The Dice coefficient is a set similarity measurement function, which is usually used to calculate the similarity between two samples, and its value range is [0,1]. The inclusion of $\left|\text{y}\cap \hat{\text{y}}\right|$ is real labels, and predicting the intersection between |y| and $\left|\hat{\text{y}}\right|$ indicates the number of elements in y and $\hat{\text{y}}$, respectively; among them, the coefficient of molecules is 2 because there is a common element in the denominator between the repeated calculation of y and $\hat{\text{y}}$

The loss function (Dice loss) is formulated according to the Dice coefficient because the real goal of segmentation is to maximize the degree of overlap between the real tag and the predicted one, that is, the similarity. However, when the Dice loss is used, there is severe shock when positive samples are generally small targets. In the case of only the foreground and background, once some pixels of small targets are predicted incorrectly, the loss value will change significantly, leading to a drastic gradient change. In the extreme case, it can be assumed that only one pixel is a positive sample. If the prediction of this pixel is correct, the prediction results of the other pixels will be ignored, and the loss is always close to 0. The prediction error causes the loss to approach 1. For the cross-entropy loss (CE loss) function, CE is a proxy form, and it is easy to maximize optimization in the network by virtue of its characteristics, which averages the value as a whole. Therefore, the loss function adopted in our experiment is to add CE loss based on the Dice loss. This can compensate for some deficiencies in the Dice loss (Li et al., 2020). The calculation formula is as follows:

Dice⁢loss⁢with⁢CE

(5) $$=1-\frac{2\,\left|y\,\cap \hat{y}\right|}{\left|y\right|+\left|\hat{y}\right|}-\left[y\,\text{log}\,\hat{y}+\left(1-y\right)\,\text{log}\,\left(1-\hat{y}\right)\right]$$

### Model Accuracy on Polyp Datasets

This section discusses an experiment that was conducted on a dataset of polyps. In order to better verify the effectiveness of our proposed model on the CT images of polyp tumor lesions, we determine the effect on polyp segmentation. We compared popular medical image segmentation semantic segmentation models: UNet (Ronneberger et al., 2015), UNet++ (Zhou et al., 2018, 2020), UNet+++ (Huang et al., 2020), U^2^Net (Qin et al., 2020), and PraNet (Fan et al., 2020), and we compared three general semantic segmentation models: PspNet (Zhao et al., 2017), Deeplabv3+ (Chen et al., 2018), and FCN8 (Long et al., 2015). To increase the reliability of our model, we added three new semantic segmentation networks: OcrNet (Yuan et al., 2020), DnlNet (Yin et al., 2020), and PointRend (Kirillov et al., 2019). For the experiment, the backbone of the model chooses the VGG16 (Simonyan and Zisserman, 2014) network as the comparison model. On the validation set data, the accuracy was analyzed based on two commonly used semantic segmentation evaluation indexes, mIOU and Dice loss with CE.

**FIGURE 5:**
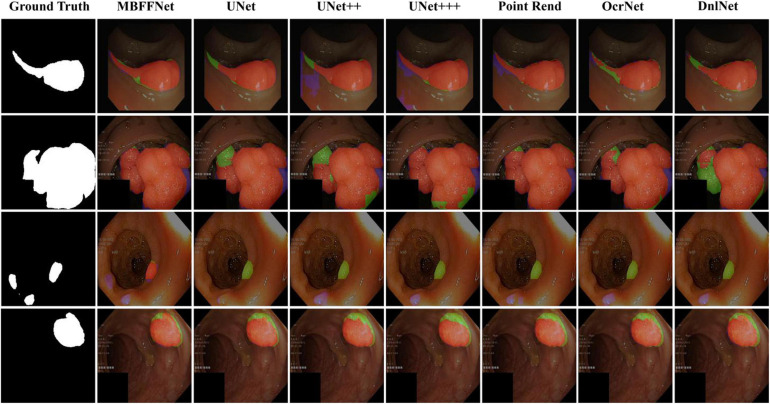
Comparison of model effect. The red represents True Positive (TP), indicating that the predicted polyp area is actually a polyp area. Blue represents False Positive (FP), indicating that the predicted polyp area is actually a non-nuclear area. The green represents FN (False Negative), which means that the predicted polyp area is actually a polyp area.

We randomly selected four test images from different angles and analyzed our model using multiple contrast models. The segmentation results are shown in Figure 5. The results of PspNet (Zhao et al., 2017), Deeplabv3+ (Chen et al., 2018), and FCN8 (Long et al., 2015), which are three general semantic segmentation models on the dataset segmentation effect, are poorer, produce serious false identification, and cannot effectively segment the region and segment the area completely, although the PraNet (Fan et al., 2020) effect is better; however, because its detection speed is much slower than MBFFNet, it does not have practical application value and is not suitable for rendering displays in the four models. As can be seen from the figure, UNet (Ronneberger et al., 2015), UNet++ (Zhou et al., 2018, 2020), U^2^Net (Qin et al., 2020), and UNet+++ (Huang et al., 2020) all segment relatively good areas and can segment the contour of the area in which the polyp is located, but the precise boundary of the polyp cannot be obtained. There are some FN pixels, especially for small polyps, and the segmentation effect of MBFFNet is obviously better than that of the other models. OcrNet (Yuan et al., 2020), DnlNet (Yin et al., 2020), and PointRend (Kirillov et al., 2019) are semantic segmentation networks, but although they show relatively excellent performance, they cannot be properly segmented in the third line of small colonoscopy images, resulting in their omission. In this study, the multiple branches feature fusion network MBFFNet is compared with the multiple model above, although it significantly reduces the number of calculations and increases the detection speed; however, because of the way in which multi-branch feature fusion is used, even small polyps in segmentation, it still makes good corresponding image edges and accurately determines the image boundary. Therefore, the MBFFNet is more effective for segmenting polyps.

As shown in Table 2, the evaluation index shows that the polyp divides the dataset on the test set, multiple-branch fusion network MBFFNet mIOU above LinkNet (Chaurasia and Culurciello, 2018), PspNet (Zhao et al., 2017), Deeplabv3+(Chen et al., 2018) general semantic network segmentation, segmentation, and medical UNet (Ronneberger et al., 2015), and the optimization model of the polyp has the same order of magnitude. Image segmentation results in a reduction in the number of calculations. The model precision does not decrease, and it can be seen that the model reduces the UNet (Ronneberger et al., 2015) redundancy phenomenon, making the model more efficient. However, in the evaluation index of Dice loss with CE, the loss value of the MBFFNet is slightly higher than that of medical networks such as UNet (Ronneberger et al., 2015), and it is much lower than that of networks such as LinkNet (Chaurasia and Culurciello, 2018). OcrNet (Yuan et al., 2020), DnlNet (Yin et al., 2020), and PointRend (Kirillov et al., 2019), which are the latest semantic segmentation networks, and they show very good performance in general semantic segmentation and show much better segmentation performance than FCN8 (Long et al., 2015), Deeplabv3+ (Chen et al., 2018), and PspNet (Zhao et al., 2017) for the colonoscopy segmentation dataset. However, because they focus more on semantic segmentation in common scenes, the segmentation effect on colonoscopy was lower than that of our proposed model and other medical image segmentation networks. This shows that the optimization of the model did not significantly affect the accuracy. It can be seen that the MBFFNet reduces redundancy in polyp segmentation, while ensuring that the accuracy does not change significantly.

**TABLE 2:** Evaluation index of polyp segmentation mIOU, *F*-score, and Dice loss with CE.

**Model**	**mlOU**	***F*-score**	**Dice loss with CE**
UNet (Ronneberger et al., 2015)	0.8883	0.9354	0.1719
LinkNet (Chaurasia and Culurciello, 2018)	0.8711	0.9238	0.1911
U^2^Net (Qin et al., 2020)	0.8950	0.9398	0.1528
UNet++ (Zhou et al., 2018, 2020)	0.8895	0.9364	0.1642
UNet+++ (Huang et al., 2020)	0.8831	0.9312	0.1827
PraNet (Fan et al., 2020)	0.9347	0.9612	0.1012
PspNet (Zhao et al., 2017)	0.8612	0.8972	0.2453
Deeplabv3+ (Chen et al., 2018)	0.8452	0.8872	0.3214
FCN8 (Long et al., 2015)	0.8563	0.8945	0.2752
DnlNet (Yin et al., 2020)	0.8657	0.9143	0.2064
OcrNet (Yuan et al., 2020)	0.8801	0.9210	0.1953
PointRend (Kirillov et al., 2019)	0.8585	0.9074	0.2153
MBFFNet	0.8952	0.9450	0.1602

### Parameter Number Verification

To better verify whether our model reduces the redundancy of the feature map and the number of parameters and flops of the model, we calculated the number of parameters and flops of the MBFFNet and LinkNet (Chaurasia and Culurciello, 2018), FCN8 (Long et al., 2015), U^2^Net (Qin et al., 2020), UNet++ (Zhou et al., 2018, 2020), UNet+++ (Huang et al., 2020), PspNet (Zhao et al., 2017), and Deeplabv3+ (Chen et al., 2018). To better compare the differences between the model parameters and the number of computations, VGG16 (Simonyan and Zisserman, 2014) was used as the backbone for all semantic segmentation networks, and the same settings were used in all comparison experiments.

The number of parameters of the model mainly depends on the number of calculations of each convolution kernel in each convolution layer. Here, the size of each convolution kernel is k_*w*_ × k_*h*_, the size of the input feature graph is c^*i*^, and the number of convolution kernels is the number of channels of the output feature graph, which is *c*^*o*^. Therefore, the calculation formula for the number of parameters at each convolution layer is as follows:

(6) $$P\,a\,r\,a\,m=c^{i}\,c^{o}\,k_{w}\,k_{h}$$

The computation of the model is the sum of each convolution layer. The number of calculations of the convolutional layer is determined by the number of calculations of the convolutional kernel in each sliding window and the overall sliding duration. In each sliding window, the number of calculations of the convolution operation is approximately $c^{i}k_{w}k_{h},l_{w}^{o}l_{h}^{o}$ is the size of the output feature graph, and the number of sliding times of the convolution kernel is the number of data of the output feature graph, that is, $c^{o}l_{w}^{o}l_{h}^{o}$, so the overall number of calculations is:

(7) $$F\,l\,o\,p\,s=c^{i}\,c^{o}\,l_{w}^{o}\,l_{h}^{o}\,k_{w}\,k_{h}$$

Using the above formula, the number of parameters in the MBFFNet and the comparison model with flops are shown in Table 3. As can be seen in the table, our MBFFNet was compared with UNet (Ronneberger et al., 2015) because of the complex model structure. MBFFNET on FLOPS reduced to 26.79% of UNET’s FLOPS; compared with U2Net (Qin et al., 2020), the quantity decreased to 24.67%, and flops to 39.51%. The results were analyzed and compared with the UNet (Ronneberger et al., 2015) model, multiple branching feature fusion network, and there was a significant reduction in the number of parameters of the model and the flop count, decreasing to a certain extent the redundancy of the model. Compared with other networks, FCN8 (Long et al., 2015) and other classical semantic segmentation networks fail to meet the requirements with respect to both precision and number of parameters. OcrNet (Yuan et al., 2020), PointRend (Kirillov et al., 2019), and DnlNet (Yin et al., 2020) have improved their accuracy, but their very high flop count requires extremely high configurations to achieve excellent performance, and they can only be applied to workstations and other environments in the future. In addition, to more comprehensively show the light weight and popularity of our model, we added the convergence time of the training model to the evaluation index of the model. It can be seen that although our model did not achieve the fastest convergence, its training time was much lower than that of UNet (Ronneberger et al., 2015), UNet++ (Zhou et al., 2018, 2020), and other networks.

**TABLE 3:** Analysis of the number of parameters and the number of calculation.

**Model**	**Training time (h)**	**Param (M)**	**Flops (B)**
UNet (Ronneberger et al., 2015)	12	24.89	56.33
LinkNet (Chaurasia and Culurciello, 2018)	3	11.53	1.23
U^2^Net (Qin et al., 2020)	18	96.25	40.24
UNet++ (Zhou et al., 2018, 2020)	20.5	36.16	135.24
UNet+++ (Huang et al., 2020)	16	18.27	211.09
PraNet (Fan et al., 2020)	13	16.16	20.37
PspNet (Zhao et al., 2017)	11.5	15.11	25.57
Deeplabv3+ (Chen et al., 2018)	16.5	134.27	27.78
FCN8 (Long et al., 2015)	78.5	30.34	6390
DnlNet (Yin et al., 2020)	15.5	50.13	50110
OcrNet (Yuan et al., 2020)	5	70.35	40530
PointRend (Kirillov et al., 2019)	37.5	47.69	14640
MBFFNet	5.5	23.74	15.09

**FIGURE 6:**
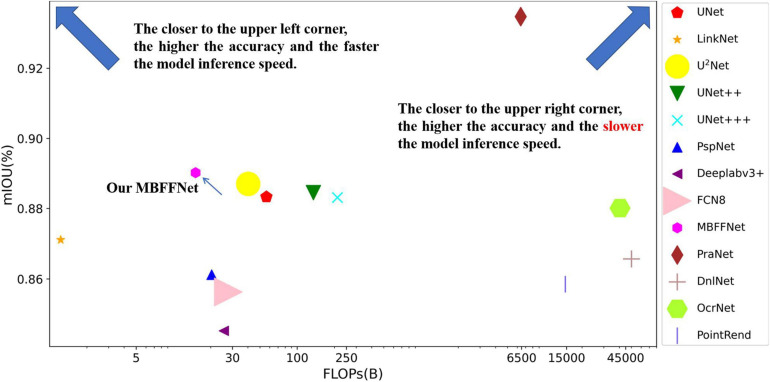
Comparison between the accuracy of different models and flop count.

To obtain a more intuitive understanding of the effects of different models, we used the flop count as the abscissa and mIOU as the ordinate, and we built a coordinate graph with the number of parameters to show the size of the model, as shown in Figure 6. From Figure 6, we observe that when the model is closer to the upper left corner, the model has a higher mIOU and a lower flop count. Although PraNet (Fan et al., 2020) possesses excellent mIOU precision, the high flop model in terms of the comprehensive income ratio is not ideal; further, although LinkNet (Chaurasia and Culurciello, 2018) has a very low flow count, the model does not have satisfactory accuracy and cannot meet the precision requirements of medical treatment, so it cannot be applied to health care.

### Real-Time Analysis of the Model

To verify that the detection rate of our model is improved when the number of parameters and number of calculations are significantly decreased, images with sizes of 256 × 256 and 64 × 64 are selected for experiments, and it is determined whether the model can meet the application standards in different computing resource environments. According to the sales data, we choose mainstream graphics cards currently on the market. GTX1060 represents the graphics card having a midrange productivity, which is the one with the highest production and the widest coverage at present. The 2060s is the midrange and top end graphics card and is the one expected to be most in use in the next 20 years. To meet the requirements of our model, it can be used in a wider range of medical environments worldwide to effectively prevent colorectal cancer and accurately separate polyps and adenomas. To test the actual operation effect of MBFFNet and considering the equipment environment in economically underdeveloped areas, we added the R5-3600 with an AMD platform and the I7-8750H CPU environment with an Intel platform, which are commonly used at present. In addition, considering that our proposed model will be applied on a large scale in medical environments, we did not choose traditional segmentation networks with poor segmentation results, such as Deeplabv3+ (Chen et al., 2018), PspNet (Zhao et al., 2017), and LinkNet (Chaurasia and Culurciello, 2018); nor did we choose PraNet (Fan et al., 2020) with poor real-time performance to conduct related experiments.

First, we selected a common medical image size of 256 × 256 as a test, and the test results are presented in Table 4. It can be seen that at 256 × 256, our model runs much faster in the CPU environment than other U-shaped networks; at its actual running speed, FPS is 100% higher than UNet (Ronneberger et al., 2015), UNet++ (Zhou et al., 2018, 2020), etc. In a GPU environment, the actual segmentation approaches 30 FPS, even on today’s midproductivity graphics cards; in real life, 30 FPS can achieve a smoother detection effect to the naked eye to meet the real-time requirements. However, other semantic segmentation models with better medical segmentation effects cannot meet the real-time requirements. Although LinkNet (Chaurasia and Culurciello, 2018) has an excellent actual operating performance, its segmentation performance fails to meet the precision requirements. In accuracy verification, the LinkNet model (Chaurasia and Culurciello, 2018) cannot effectively segment the polyp boundary.

**TABLE 4:** 256 × 256 polyp image segmentation FPS.

**Model**	**AMD**	**Inter**	**2060Super**	**1060**
Unet (Ronneberger et al., 2015)	4	3	45	21
LinkNet (Chaurasia and Culurciello, 2018)	19	16	115	88
U^2^Net (Qin et al., 2020)	2	2	23	14
UNet++ (Zhou et al., 2018, 2020)	2	2	22	10
UNet+++ (Huang et al., 2020)	2	1	16	8
MBFFNet	8	7	55	28

*The image size is 256 × 256. AMD represents the FPS test on the CPU of the AMD platform (R5-3600), Inter represents the FPS test on the CPU of Intel platform (I7-8750H), 2060Super represents the FPS test in the GPU environment of the 2060Super graphics card, and 1060 represents the FPS test in the GPU environment of the RTX1060 graphics card.*

Subsequently, we conducted FPS test experiments on 64 × 64 images, and the experimental results are listed in Table 5. In the 64 × 64 image, our model can meet the real-time test requirement of 30 FPS even in a CPU environment, and the actual running fluency FPS is higher than that of other medical image segmentation networks. Thus, it can be seen that in existing common computer resources equipment, MBFFNet can meet the requirements of real-time observation of medical observation, even in economically underdeveloped areas. For low computer resources, it is seen that even in the case of infrequently used graphics resources configuration, our proposed model can also guarantee the real-time segmentation of polyps.

**TABLE 5:** FPS segmentation of 64 × 64 polyp images.

**Model**	**AMD**	**Inter**	**2060Super**	**1060**
Unet (Ronneberger et al., 2015)	20	19	152	90
LinkNet (Chaurasia and Culurciello, 2018)	84	68	138	141
U^2^Net (Qin et al., 2020)	13	14	31	23
UNet++ (Zhou et al., 2018, 2020)	10	11	98	55
UNet+++ (Huang et al., 2020)	9	9	90	68
MBFFNet	33	31	163	112

*The image size is 64 × 64. AMD stands for the FPS test on AMD CPU (R5-3600), Intel stands for the FPS test on an Intel CPU (I7-8750H), 2060Super stands for the FPS test in the GPU environment on a 2060Super graphics card, and 1060 stands for FPS test in the GPU environment on an RTX1060 graphics card.*

Based on the experiment results, it can be seen that owing to the advantages of low flop count, our model displays excellent real-time performance in an environment with low computer resources, while the advantages of our model are very significant in environments with lower computer resources. Under the current computer resources, our model MBFFNet has been able to deal with a variety of different conditions of accurate basic real-time polyp segmentation and achieved a relatively good effect.

### Model Generalization Experiment

**FIGURE 7:**
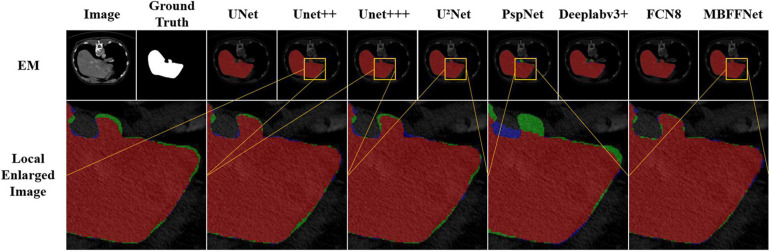
Segmentation effect of liver lesions.

For all of the experiments in this section, we chose the same experimental environment and image processing method as the polyp segmentation dataset in *Dataset*. The final evaluation indexes mIOU, *F* score, and Dice loss with CE were also evaluated based on validation set data. We chose U^2^Net (Qin et al., 2020), UNet++ (Zhou et al., 2018, 2020), and UNet+++ (Huang et al., 2020) as the semantic segmentation models for medical images; PraNet (Fan et al., 2020) as the semantic segmentation model for polyps; and PspNet (Zhao et al., 2017), Deeplabv3+ (Chen et al., 2018), and FCN8 (Long et al., 2015) as the comparison model for the experiment. For the demonstration, we selected the test sample for liver lesion segmentation, and the sample segmentation image is shown in Figure 7. It can be seen that, compared with other models, MBFFNet retains the edge feature information better, which makes the boundary of liver lesion segmentation clearer and more accurate, and ensures the accuracy of medical images.

**FIGURE 8:**
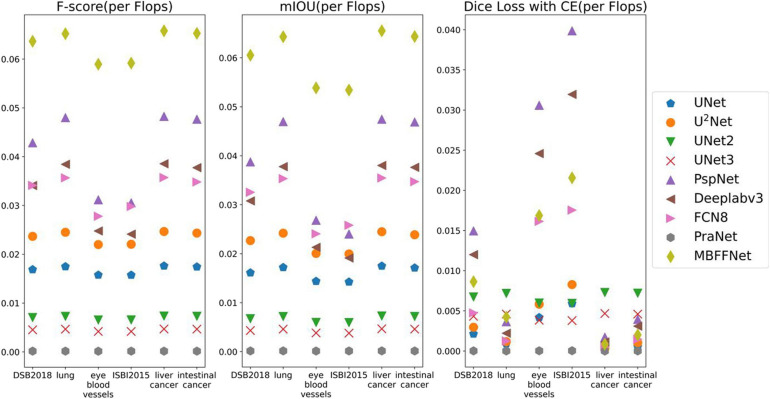
Comparison between the accuracy of different models and Flop count.

According to the analysis of the experimental results, similar to the results of colonoscopy segmentation, our model is better than PraNet (Fan et al., 2020), Deeplabv3+ (Chen et al., 2018), FCN8 (Long et al., 2015), and other general semantic segmentation models in various medical image segmentation datasets, but it is slightly better than UNet (Ronneberger et al., 2015), UNet++ (Zhou et al., 2018, 2020), and U^2^Net (Qin et al., 2020) and basically equal to UNet+++ (Huang et al., 2020). The segmentation results of the model are worse than those of PraNet (Fan et al., 2020). As these medical models can all achieve good segmentation effects, mIOU, *F* score, Dice loss with CE, and other indicators show excellent effects in intestinal cancer, liver cancer, DSB2018, lung, and other datasets, with little difference. In the face of a more complex medical image segmentation environment, for example, only in the eye blood vessels and ISBI2015 datasets can PraNet (Fan et al., 2020) show relatively good results. It can be seen that the PraNet (Fan et al., 2020) model can achieve a good segmentation effect in a very complex segmentation environment, but its extremely large flop count makes it impossible to carry out an effective real-time segmentation model in a generally productive equipment. However, our MBFFnet model retains edge feature information owing to multi-branch feature fusion. In most circumstances, it can achieve excellent segmentation results and has good generalization ability, which is sufficient to deal with most of the image segmentation, and because our model with network model structure is compact and lightweight, it enables very convenient deployment in most of medical environments, lesion image segmented (see the Appendix for detailed experimental results in Tables A1–A3). Because the ultimate purpose of this study is to find a network that can be applied in practice and that considers both speed and precision, it is not ideal to talk about precision without speed alone. Therefore, the ratio of mIOU, *F* score, and Dice loss with CE to flops was taken as the index of the new measurement model. It can be seen from mIOU (per flops) and *F* score (per flop) that our model has the highest return under the same computing resources (the higher the better), whereas the loss indicator indicates a faster and more stable convergence (the lower the better). The effect diagram is shown in Figure 8.

## Conclusion

In this article, an MBFFNet is proposed to achieve the accurate and real-time segmentation of liver lesion images. A U-shaped structure such as UNet is used to gradually fuse shallow features with high-dimensional features. The method of superposition of feature graphs used by UNet is abandoned in the process of feature fusion, but the multiplication of feature graphs is chosen for feature fusion. A feature map with five branches is used, and then a pyramid feature map similar to PspNet is used to fuse the feature as a supplementary feature of the feature information. Finally, the two groups of features are fused to obtain the final segmentation result, and the experimental results show that the algorithm in the segmentation polyp area achieved the same results as the UNet segmentation results regardless of the polyp area size. In addition, it can complete the segmentation edge details such as features, get a better segmentation effect, and significantly reduce the network number and number of calculations, and it improved the real-time performance of the polyp of semantic segmentation model segmentation; at the same time, the segmentation experiments on other medical images show that MBFFNet has good robustness in medical image segmentation.

## Data Availability Statement

The original contributions presented in the study are included in the article/supplementary material, further inquiries can be directed to the corresponding author/s.

## Author Contributions

HS: writing–editing, conceptualization, investigation, and model validation. BL: project administration, writing–editing, and model improvement. XH: writing–original draft and visualization. JL: formal analysis and project improvement. KJ: writing–review. XD: funding acquisition and methodology. All authors contributed to the article and approved the submitted version.

## Conflict of Interest

The authors declare that the research was conducted in the absence of any commercial or financial relationships that could be construed as a potential conflict of interest.

## Appendix

**TABLE A1:** mIOU evaluation index of multi-class medical image segmentation.

**Model**	**mIOU**					
	**DSB2018**	**Lung**	**Eye blood vessels**	**ISBI2015**	**Liver cancer**	**Intestinal cancer**
UNet (Ronneberger et al., 2015)	0.9078	0.9691	0.8106	0.8020	0.9854	0.9641
LinkNet (Chaurasia and Culurciello, 2018)	0.8983	0.9166	0.7457	0.7711	0.9803	0.9279
U^2^Net (Qin et al., 2020)	0.9126	0.9732	0.8070	0.8031	0.9854	0.9609
UNet++ (Zhou et al., 2018, 2020)	0.9108	0.9697	0.8083	0.8023	0.9849	0.9733
UNet+++ (Huang et al., 2020)	0.9134	0.9715	0.8077	0.7995	0.9858	0.9707
PraNet (Fan et al., 2020)	0.9453	0.9897	0.8762	0.8862	0.9903	0.9801
PspNet (Zhao et al., 2017)	0.7892	0.9568	0.5464	0.4891	0.9667	0.9551
Deeplabv3+ (Chen et al., 2018)	0.7871	0.9661	0.5449	0.4890	0.9721	0.9623
FCN8 (Long et al., 2015)	0.9041	0.9815	0.6687	0.7172	0.9853	0.9645
MBFFNet	0.9132	0.9704	0.8127	0.8061	0.9884	0.9709
						

**TABLE A2:** Multi-class medical image segmentation *F*-score evaluation index.

**Model**	***F*-score**					
	**DSB2018**	**Lung**	**Eye blood vessels**	**ISBI2015**	**Liver cancer**	**Intestinal cancer**
UNet (Ronneberger et al., 2015)	0.9502	0.9842	0.8872	0.8864	0.9926	0.9815
LinkNet (Chaurasia and Culurciello, 2018)	0.9446	0.9803	0.8379	0.8657	0.9900	0.9616
U^2^Net (Qin et al., 2020)	0.9527	0.9864	0.8846	0.8873	0.9926	0.9798
UNet++ (Zhou et al., 2018, 2020)	0.9519	0.9845	0.8855	0.8865	0.9923	0.9863
UNet+++ (Huang et al., 2020)	0.9532	0.9855	0.8851	0.8846	0.9928	0.9850
PraNet (Fan et al., 2020)	0.9732	0.9912	0.9213	0.9274	0.9912	0.9883
PspNet (Zhao et al., 2017)	0.8729	0.9778	0.6350	0.6223	0.9828	0.9712
Deeplabv3+ (Chen et al., 2018)	0.8714	0.9827	0.6337	0.6170	0.9857	0.9653
FCN8 (Long et al., 2015)	0.9478	0.9906	0.7722	0.8278	0.9925	0.9671
MBFFNet	0.9604	0.9839	0.8895	0.8928	0.9926	0.9851
						

**TABLE A3:** Dice loss with CE evaluation index for multi-class medical image segmentation.

**Model**	**Dice Loss with CE**					
	**DSB2018**	**Lung**	**Eye blood vessels**	**ISBI2015**	**Liver cancer**	**Intestinal cancer**
UNet (Ronneberger et al., 2015)	0.1264	0.0548	0.2222	0.3310	0.0146	0.0377
LinkNet (Chaurasia and Culurciello, 2018)	0.1423	0.0714	0.3258	0.3822	0.0215	0.0779
U^2^Net (Qin et al., 2020)	0.1191	0.0471	0.2344	0.3327	0.0148	0.0411
UNet++ (Zhou et al., 2018, 2020)	0.1213	0.0547	0.2248	0.3202	0.0151	0.0276
UNet+++ (Huang et al., 2020)	0.1199	0.0514	0.2351	0.3331	0.0144	0.0307
PraNet (Fan et al., 2020)	0.0921	0.0321	0.1453	0.2145	0.0101	0.0219
PspNet (Zhao et al., 2017)	0.3046	0.0743	0.6231	0.8119	0.0351	0.0801
Deeplabv3+ (Chen et al., 2018)	0.3068	0.0565	0.6285	0.8169	0.0290	0.0792
FCN8 (Long et al., 2015)	0.1318	0.0350	0.4485	0.4874	0.0145	0.0407
MBFFNet	0.1303	0.0638	0.2545	0.3254	0.0130	0.0303
